# Supplementary material for: Simulated patient systems powered by large language model-based AI agents offer potential for transforming medical education
Source: Commun Med (Lond). 2025 Dec 19;6:27. doi: 10.1038/s43856-025-01283-x (PMC12808140; doi:10.1038/s43856-025-01283-x)
Supplement: Supplementary file 1 — Supplementary Information [file 43856_2025_1283_MOESM1_ESM.pdf]

Supplementary Information

Supplementary Methods

Supplementary Method 1. Data Annotation and Evaluation Details

We used the Doccano annotation interface (detailed in **Supplementary Fig. 6**) to annotate the gold-standard labeled dataset, where 100 cases were selected and six physicians independently labeled the medical entities of interest, including symptom, medical history, vitals, allergies, family history, and social history. The F1 score of 0.79 provides a balance between precision and recall, and evaluating it at the span level takes into account the exact start and end positions of entities. This makes span-level evaluation more rigorous than simple token-based assessments, as it ensures that both the correct entity type and its precise boundaries within the text are accurately identified. Finally, separate physicians, who did not participate in the data labeling process before, reconciled the differences between the labeling.

For the F1 score, it is based on True Positive Rate, False Positive Rate, Precision, and Recall as follows:

- **True Positive Rate**: TP/(TP + FN)
- **False Positive Rate**: FP/(TN + FP)
- **Precision**: TP/(TP + FP)
- **Recall**: TP/(TP + FN)
- **F1**: (2*Precision*Recall)/(Precision+Recall)

Here, we specify the in-context definitions of TP, FP, TN, and FN:

- TP: Number of instances where both LLM and gold-standard data agree a medical entity is present.
- FP: Number of instances where LLM indicates a medical entity is present, but gold-standard data shows no medical entity.
- TN: Number of instances where both LLM and gold-standard data agree no entity is present.
- FN: Number of instances where LLM indicates no medical entity, but gold-standard data shows a medical entity is present.

For the second dataset, the Medical QA dataset, labeled by NLP researchers, an example for a symptom-related question is: "what is the duration of the symptom 'chest pain'?" A subset of the questions is focused on complete set retrieval, e.g., "What are all the symptoms that the patient with SUBJECT_ID = 22265 and HADM_ID = 147802 have?" These questions test how accurate, readable, robust, and stable the AIPatient system is, with a variety of input formats, including generic and iterative paraphrasing on the same set of questions.

Using the Medical QA dataset, we evaluate QA accuracy, Readability, Robustness, and Stability. For the later three aspects, we prompt the Rewrite Agent to write the answer in the context of the question. This step mimics the Rewrite process in the AIPatient framework, where the Rewrite Agent formulates natural language responses to user's questions based on the Cypher query results. For readability, we calculate the Flesch Reading Ease and Flesch-Kincaid Grade Level for each of the rewritten responses.

Robustness is another critical component of the trustworthiness of AIPatient, reflecting the system's reliability and consistency across varied inputs. To evaluate the robustness of AIPatient, we employ a methodology centered around the resilience of the system to changes in the phrasing of questions and assess the system robustness. This paraphrasing mimics the real-world scenario where different users may phrase the same clinical question in various ways.

For stability, the Rewrite Agent’s task is to adjust the responses generated by the system to reflect the specific personality traits of the simulated patient while answering questions from the QA set. This involves altering the tone, style, and certain expressions in the answer to align with the given personality, without altering the core medical information. Finally, researchers manually review the outputs to ensure that all personality-infused rewrites maintain the core information from the original answers. The primary goal is twofold:

1. To confirm that the inclusion of personality traits in the rewrites does not lead to a loss of essential information compared to rewrites without personality traits;
2. To check that there is no statistically significant difference in the accuracy of information across different personality groups.

Supplementary Method 2. LLM Parameter Details

We used 11 different LLM for AIPatient performance evaluation tasks. For each LLM, we set the values of the parameters to max_tokens_to_sample = 4096, which limits the length of input to 4096 tokens, and temperature = 1, which sets relative high diversity of model output. The model names per tasks are as follows:

1. LLM Selection and knowledgebase Validity (NER)
    a. Closed Source Models: Claude-3 haiku, Claude-3 sonnet, Claude-3.5 sonnet, Claude-4-sonnet, Claude-4-opus, GPT-3.5 turbo, GPT-4o, and GPT-4 turbo
    b. Open Source Models: Deepseek-v3: 671b, Qwen-4: 32b, Llama-3: 17b
2. QA Conversation Accuracy Ablation Study
    a. Closed Source Models: Claude-3 haiku, Claude-3.5 sonnet, Claude-4-sonnet, Claude-4-opus, GPT-3.5 turbo, GPT-4o, and GPT-4 turbo
    b. Open Source Models: Deepseek-v3: 671b, Qwen-4: 32b, Llama-3: 17b
3. Readability: GPT-4 turbo;
4. Robustness (system): GPT-4 turbo, and ;
5. Stability (personality): GPT-4 turbo.

Supplementary Method 3. Prompt Engineering Strategy

We enable these strategies when constructing the prompts for optimizing the performance of various agents within the system. These strategies support good interaction and accurate retrieval, generation and simulation of patients:

1. **Role specification** [1]**:** Role specification is often used to set context for the task at hand. In the case of biomedical domains, defining the model's role as a biomedical researcher helps guide the LLM to focus on relevant terms and relationships specific to that domain. Using predefined roles aligns with studies showing that role-specific context boosts performance in biomedical text mining and entity recognition.
2. **Few-shot** [2,3]: We include a few examples in the prompts, which allow the models to generalize effectively by drawing on prior knowledge. This approach significantly reduces the need for large volumes of annotated medical data while still maintaining high accuracy in tasks like entity recognition and relationship extraction.
3. **XML-style tag** [4]: To improve entity extraction and relationship mapping, we employ XML-style tagging in our prompts. This structured format ensures that the output is both readable and easily processed.

Supplementary Method 4. AIPatient System Implementation Details
The AIPatient system was implemented in Python (v3.9.16), combining large language model APIs, graph database interfaces, and prompt engineering modules.

1. **Knowledge Graph Construction**. Discharge summaries were first parsed using LLM-based Named Entity Recognition (NER) via OpenAI's GPT-4 Turbo through Azure API, complying with PhysioNet's privacy policy. Entity types include symptoms, vitals, allergies, medical history, family and social history, and were structured as triples (e.g., Patient–HAS_SYMPTOM→Symptom) stored in Neo4j (AuraDB version 5) [5]. We used Cypher query language to interact with the KG via the neo4j Python driver.
2. **Agentic Workflow Execution.** The Reasoning RAG workflow was orchestrated using a modular Python class structure with six sequential agents. Each agent is a Python module that calls an LLM through a designated wrapper, with JSON-formatted inputs and system prompts adapted from Supplementary Methods.
3. **Conversation Management**. Multi-turn conversations are tracked using a conversation history manager, implemented as a stateful dictionary updated after each user-agent interaction. This history is embedded in prompts to simulate memory and personality continuity, enabling agents like the Rewrite and Summarization Agent to respond consistently across turns.
4. **Evaluation Pipelines**. QA accuracy, robustness, and stability were evaluated via automated scripts that parse GPT outputs and compare them against gold-standard answers. Readability metrics were calculated using textstat library, and statistical tests (t-tests, ANOVA) were performed using scipy.stats.
5. **Infrastructure and API Use**. Model inference was performed via cloud-hosted APIs or local deployment. Specifically, we used Azure OpenAI endpoints for GPT and Deepseek models, and Amazon Bedrock for Claude models, which is in full compliance with MIMIC-III data-sharing restrictions. Two other open-source language models—Qwen3-32B, LLaMA3.3-70B—were deployed via the Ollama framework. Each model is specified using a Modelfile that defines the base model, system prompts, and configuration options. Ollama provides isolated, GPU-accelerated execution through an optimized backend and initializes models via the CLI (ollama run <model_name>). All models ran in their original open-source form without fine-tuning. Deployment is performed on an Ubuntu 20.04.6 LTS system with two AMD EPYC 9654 96-core processors, 256 GB RAM, and 8 NVIDIA RTX A6000 GPUs (48 GB VRAM each). No patient data was uploaded to public endpoints. Local computation was limited to orchestration scripts, evaluation pipelines, and knowledge graph management. The Neo4j graph database ran in a cloud-hosted AuraDB instance, with saved snapshots for consistency and backup.

Supplementary Method 5. Operational Flow of the AIPatient Multi-Agent System

The Retrieval Stage begins with the Retrieval Agent that selects a subset of relevant nodes and edges of the AIPatient KG, based on the natural language query. In parallel, the Abstraction Agent [6] in the Reasoning Stage simplifies the specific or detailed user natural language query and paraphrases them into generalized, high-level questions. Taking the inputs from Retrieval Agent and Abstraction Agent, the KG Query Generation Agent constructs a Cypher query [6], which is a declarative language particularly designed for Neo4j databases that can efficiently query and manipulate graph data through nodes, relationships, and properties. KG Query in Cypher is preferred over direct document RAG approaches. The queries can navigate complex graph structures, extracting rich and connected insights that are critical in understanding multidimensional relationships among medical entities. For example, symptoms and diseases are harder to capture with linear document-based retrieval methods. The final output of this stage is the Cypher query-retrieved information, such as symptom or disease intensity.

Next, the Checker Agent in the Reasoning Stage makes a decision of whether the retrieved information and the medical investigation inquiry aligns – if the Checker Agent approves the results (outputs "Yes"), the workflow proceeds to the Generation stage; if not (outputs "No"), the Checker Agent rephrases the natural language query based on the Conversation History and prompts the KG Query Generation Agent to regenerate the Cypher query. The checking process repeats for three times, and if no satisfactory information is retrieved after three iterations, the Generation Stage is skipped and the simulated patient outputs "I don't know".

The Generation Stage focuses on the alignment of medical information to realistic and diverse patient behaviors in responses. The Rewrite Agent aims to translate the technical KG query results into a more accessible and understandable natural language format, taking into account the specific personality traits assigned to the patient. It simulates the patient's personality based on the Big Five personality traits model through prompting [7]. For single-round conversation, this output is passed back to the medical investigator. For multi-round conversation, the Summarization Agent then integrates the rewritten results and conversation and accordingly updates the conversation history.

Supplementary Method 6. NER Prompt Engineering Details

AIPatient KG is constructed from the medical entities (nodes) and relationships (edges) extracted from the unstructured clinical notes. Relationships illustrate how entities are interconnected, such as “patient has certain symptoms” and “disease has specific duration.” These extractions are based on an LLM powered NER approach, using GPT-4 model. In particularly, we use prompts with the following characteristics:

You are a biomedical and AI researcher.
Please format the allergies and adverse reactions from the following text. Don't include any adjectives or adverbs.
If the text shows one medication name or a list of medication names, you should consider it or them as allergies.
If there are no allergies / medications or patient explicitly stated they don't have any allergies, output <N/A>.
If there are multiple are identified, separate with ";"; if none are identified, output <N/A>
All the extracted information should be in the exact wording as the text; do not paraphrase.
Output_format: "Enclose your output within <root><root> tags. For example: <allergies>{'Answer': penicillins; percocet; morphine}<allergies>
The text is: [Abbreviated for simplicity]

Supplementary Method 7. AIPatient KG Schema

The full schema of the AIPatien KG is as follows. This schema is included in the prompt to allow agents to effectively query and retrieve relevant EHR data from the knowledge graph.

```
### Node Properties
- Patient: SUBJECT_ID, GENDER, AGE, ETHNICITY, RELIGION, MARITAL_STATUS
- Admission: HADM_ID, DURATION, ADMISSION_TYPE, ADMISSION_LOCATION, DISCHARGE_LOCATION, INSURANCE
- Symptom: name
- Duration: name
- Intensity: name
- Frequency: name
- History: name
- Vital: LABEL, VALUE
- Allergy: name
- SocialHistory: description
- FamilyMember: name
- FamilyMedicalHistory: name

### Relationships
- Patient -> Admission: HAS_ADMISSION
- Patient -> History: HAS_MEDICAL_HISTORY
- Patient -> FamilyMember: HAS_FAMILY_MEMBER
- Admission -> Symptom: HAS_SYMPTOM
- Admission -> SocialHistory: HAS_SOCIAL_HISTORY
- Admission -> Allergy: HAS_ALLERGY
- Admission -> Symptom: HAS_NOSYMPTOM
- Admission -> Vital: HAS_VITAL
- Symptom -> Duration: HAS_DURATION
- Symptom -> Intensity: HAS_INTENSITY
- Symptom -> Frequency: HAS_FREQUENCY
- FamilyMember -> FamilyMedicalHistory: HAS_MEDICAL_HISTORY
```

Supplementary Method 8. Retrieval Agent Details

- conversation_history: summarized conversation history between AIPatient and user. For example: Patient ID 23709 (Admission ID 182203) initially reported black and bloody stools. The patient has a complex medical history including depression, heart and brain issues, stomach troubles, and previous falls with broken bones. When asked about allergies, the patient disclosed an allergy to SSRI medications but expressed reluctance to discuss it further. The current context relates to the patient's allergy history.
- user_query: the input query from the user. For example: “Can you tell me your symptoms?”
- schema: the general structure of the knowledge graph, including node properties and relationships, as specified in the supplementary methods of AIPatient KG Schema. For example: Patient node has properties: SUBJECT_ID, GENDER, AGE, ETHNICITY, RELIGION, MARITAL_STATUS; relationship: FamilyMember -> FamilyMedicalHistory: HAS_MEDICAL_HISTORY

```
Based on the doctor's query, first determine what the doctor is asking for. Then extract the appropriate relationship and nodes from the knowledge graph. \n
        For admissions related queries, the query should focus on "HAS_ADMISSION" relationship and "Admission" node. \n
        For patient information related queries, the query should focus on the "Patient" node. \n
        If the doctor asked about a symptom (e.g. cough, fever, etc.), the query should check if the "symptom" node and the "HAS_SYMPTOM" or "HAS_NOSYMOTOM" relationship; \n
        If the doctor asked about the duration, frequency, and intensity of a symptom, the query should first check if the symptom exist. If it exist, then check the "duration", "frequency" and "intensity" node respectively, and "HAS_DURATION", "HAS_FREQUENCY", "HAS_INTENSITY" relationship respectively. \n
        If the doctor asked about medical history, the query should check "History" node and the HAS_MEDICAL_HISTORY relationship. \n
        If the doctor asked about vitals (temperature, blood pressure etc), the query should check the "Vital" node and "HAS_VITAL" relationship. \n
        If the doctor asked about social history (smoking, alcohol consumption etc), the query should check the "SocialHistory" node and "HAS_SOCIAL_HISTORY" relationship. \n
        If the doctor aksed about family history, the query should first check the "HAS_FAMILY_MEMBER" relationship and "FamilyMember" node. Then, the query should check the "HAS_MEDICAL_HISTORY" relationship and "FamilyMedicalHistory" node associated with the "FamilyMember" node. \n

Output_format: Enclose your output in the following format. Do not give any explanations or reasoning, just provide the answer. For example:
        {{'Nodes': ['symptom', 'duration'], 'Relationships': ['HAS_SYMPTOM', 'HAS_DURATION']}}

        The natural language query is:
        {user_query}

        The previous conversation history is:
        {conversation_history}

        The Knowledge Graph Schema is:
        {schema}
```

Supplementary Method 9. Abstraction Agent Details

- user_query: the input query from the user. For example: “Can you tell me your symptoms?”
- conversation_history: summarized conversation history between AIPatient and user. For example: Patient ID 23709 (Admission ID 182203) initially reported black and bloody stools. The patient has a complex medical history including depression, heart and brain issues, stomach troubles, and previous falls with broken bones. When asked about allergies, the patient disclosed an allergy to SSRI medications but expressed reluctance to discuss it further. The current context relates to the patient's allergy history.

```
You are an AI and Medical EHR expert. Your task is to step back and paraphrase a question to a more generic step-back question, which is easier to use for cypher query generation. \n
        If the question is vague, consider the conversation history and the current context. Do not give any explanations or reasoning, just provide the answer.
        Here are a few examples: \n
        input: Do you have fevers as a symptom? \n
        output: What symptoms does the patient has? \n
        input: Is your current temperature above 97 degrees? \n
        output: What is the patient's temperature? \n

        The current conversation history is:
        {conversation_history}
        The original query is:
        {user_query}
```

Supplementary Method 10. KG Query Generation Agent Details

An example of cypher query generation prompt (with few-shot, abstraction agent, and retrieval agent input) is listed below:

- subject_id: unique patient identifier
- hadm_id: unique admission identifier
- nodes_edges: output of the Retrieval Agent, contains relevant nodes and edges for constructing the cypher query. For example: {'Nodes': ['Allergy'], 'Relationships': ['HAS_ALLERGY']}
- conversation_history: summarized conversation history between AIPatient and user. For example: Patient ID 23709 (Admission ID 182203) initially reported black and bloody stools. The patient has a complex medical history including depression, heart and brain issues, stomach troubles, and previous falls with broken bones. When asked about allergies, the patient disclosed an allergy to SSRI medications but expressed reluctance to discuss it further. The current context relates to the patient's allergy history.
- user_query: the input query from the user. For example: “Can you tell me your symptoms?”
- schema: the general structure of the knowledge graph, including node properties and relationships, as specified in the supplementary methods of AIPatient KG Schema. For example: Patient node has properties: SUBJECT_ID, GENDER, AGE, ETHNICITY, RELIGION, MARITAL_STATUS; relationship: FamilyMember -> FamilyMedicalHistory: HAS_MEDICAL_HISTORY
- abstraction_context: output of the Abstraction Agent, contains step-back context. For example, when the user asks about whether the patient has fever, the step back context is a list of all the symptoms the patient has.

```
Write a cypher query to extract the requested information from the natural language query. The SUBJECT_ID is {subject_id}, and the HADM_ID is {hadm_id}.

The nodes and edges the query should focus on are {nodes_edges} \n

Note that if the doctor's query is vague, it should be referring to the current context.\n

The Cypher query should be case insensitive and check if the keyword is contained in any fields (no need for exact match). \n

The Cypher query should handle fuzzy matching for keywords such as 'temperature', 'blood pressure', 'heart rate', etc., in the LABEL attribute of Vital nodes.\n

The Cypher query should also handle matching smoke, smoking, tobacco if asked about smoking and social history; similarly for drinking, or alcohol. \n

Only return the query as it should be executable directly, and no other text. Don't include any new line characters, or back ticks, or the word 'cypher', or square brackets, or quotes.\n

The previous conversation history is: {conversation_history}

The natural language query is: {user_query}

The Knowledge Graph Schema is: {schema}

The step back context is: {abstraction_context}

Here are a few examples of Cypher queries, you should replay SUBJECT_ID and HADM_ID based on input:\n

Example 1: To check if the patient has seizures as a symptom, the cypher query should be:
    MATCH (p:Patient {{SUBJECT_ID: 23709}})
    OPTIONAL MATCH (p)-[:HAS_ADMISSION]->(a:Admission {{HADM_ID: 182203}})-[:HAS_SYMPTOM]->(s:Symptom)
    WHERE s.name =~ '(?i).*seizure.*'
    WITH p, a, s
    OPTIONAL MATCH (p)-[:HAS_ADMISSION]->(a)-[:HAS_NOSYMPTOM]->(ns:Symptom)
```

```
        WHERE ns.name =~ '(?i).*seizure.*'
        RETURN
        CASE
            WHEN s IS NOT NULL THEN 'HAS seizure'
            WHEN ns IS NOT NULL THEN 'DOES NOT HAVE seizures'
            ELSE 'DONT KNOW'
        END AS status

Example 2: To check how long has the patient had fevers as a symptom, the cypher query should be:
        MATCH (p:Patient {{SUBJECT_ID: 23709}})
        OPTIONAL MATCH (p)-[:HAS_ADMISSION]->(a:Admission {{HADM_ID: 182203}})-[:HAS_SYMPTOM]->(s:Symptom)
        WHERE s.name =~ '(?i).*fever.*'
        WITH p, a, s
        OPTIONAL MATCH (s)-[:HAS_DURATION]->(d:Duration)
        RETURN
        CASE
            WHEN s IS NULL THEN 'DOES NOT HAVE fevers'
            WHEN d IS NULL THEN 'DONT KNOW'
            ELSE d.name
        END AS fever_duration

Example 3: To check for family history, the cypher query should be:
        MATCH (p:Patient {{SUBJECT_ID: 23709}})-[:HAS_FAMILY_MEMBER]->(fm:FamilyMember)
        OPTIONAL MATCH (fm)-[:HAS_MEDICAL_HISTORY]->(fmh:FamilyMedicalHistory)
        RETURN fm.name AS family_member, fmh.name AS medical_history
```

Supplementary Method 11. Checker Agent Details

An example of Checker Agent prompt is provided below:

- user_query: the input query from the user. For example: “Can you tell me your symptoms?”
- conversation_history: summarized conversation history between AIPatient and user. For example: Patient ID 23709 (Admission ID 182203) initially reported black and bloody stools. The patient has a complex medical history including depression, heart and brain issues, stomach troubles, and previous falls with broken bones. When asked about allergies, the patient disclosed an allergy to SSRI medications but expressed reluctance to discuss it further. The current context relates to the patient's allergy history.
- query_result: extracted result from AIPatient KG based on the user_query. For example, ['black and bloody stools', 'lightheadedness', 'shortness of breath'] for the user query: “what symptoms do you have?”

```
You are a doctor's assistant. You are recording and evaluating patient's responses to doctor's query.
    The conversation history between the doctor and patient is as follows:
    {conversation_history}
    The doctor's query is:
    {user_query}
    The query result is:
    {query_result}
    Based on the above conversation, determine if the patient's response is an appropriate answer to the doctor's query.
    If so, return 'Y' and do not return anything else; if not, rewrite the doctor's query based on the current context; only return the modified query and nothing else.
```

Supplementary Method 12. Rewrite Agent Details

This Rewrite Agent includes five broad dimensions: openness, conscientiousness, extraversion, agreeableness, and neuroticism. Each patient is assigned specific traits within these dimensions to create a realistic and relatable personality profile. For example, a patient with personality traits of “sensitive/nervous” in the neuroticism category might repeatedly ask for reassurance about their condition and treatment plan, and another with “friendly/compassionate” in the agreeableness category might be open to engaging in more detailed conversations and expressing gratitude for the care provided.

An example of Rewrite Agent prompt is provided below:

- personality: the selected Big Five personality profile. It is a combination of five dimensions including: extraversion, agreeableness, openness, conscientiousness, and neuroticism. An example of one personality profile is [practical, conventional, prefers routine, Impulsive, careless, disorganized, quiet, reserved, withdrawn, critical, uncooperative, suspicious, calm, even-tempered, secure]
- user_query: the input query from the user. For example: “Can you tell me your symptoms?”
- conversation_history: summarized conversation history between AIPatient and user. For example: Patient ID 23709 (Admission ID 182203) initially reported black and bloody stools. The patient has a complex medical history including depression, heart and brain issues, stomach troubles, and previous falls with broken bones. When asked about allergies, the patient disclosed an allergy to SSRI medications but expressed reluctance to discuss it further. The current context relates to the patient’s allergy history.
- query_result: extracted result from AIPatient KG based on the user_query. For example, ['black and bloody stools', 'lightheadedness', 'shortness of breath'] for the user query: “what symptoms do you have?”

```
You are a virtual patient in an office visit. Your personality is {personality}.
    Your conversation history with the doctor is as follows:
    {conversation_history}
    The doctor has asked about the following query, focusing on the current context (e.g. vital, symptom, or history):
    {user_query}
    The query result is:
    {query_result}
    Based on all above information, please write your response to the doctor following your personality traits. Note that if the doctor's query is vague, it should be referring to the current context.
    If the query result is empty, return 'I don't know.' DO NOT fabricate any symptom or medical history. DO NOT add non-existent details to the response. DO NOT inclue any quotes, write in first person perspective.
```

Supplementary Method 13. Summarization Agent Details

- conversation_history: summarized conversation history between AIPatient and user. For example: Patient ID 23709 (Admission ID 182203) initially reported black and bloody stools. The patient has a complex medical history including depression, heart and brain issues, stomach troubles, and previous falls with broken bones. When asked about allergies, the patient disclosed an allergy to SSRI medications but expressed reluctance to discuss it further. The current context relates to the patient's allergy history.
- query_result: extracted result from AIPatient KG based on the user_query. For example, ['black and bloody stools', 'lightheadedness', 'shortness of breath'] for the user query: "what symptoms do you have?"
- patient_response: natural language response from the simulated patient. For example: "Yes, I am over 50. But why does that matter right now? I prefer to stick to the routine questions we usually go through. And honestly, I don't see how my age is relevant to what we're discussing today. Can we move on to something more practical?"

```
You are the doctor's assistent responsible for summarizing the conversation between the doctor and the patient.
        Be very brief, include the all the conversation history, doctor and patient's query and response. The last sentence should be about the current context (e.g. vital, symptom, or history).
        Write in full sentences and do not fabricate symptoms or history.
        The previous conversation is as follows:
        {conversation_history}
        The doctor has asked about the following query:
        {user_query}
        The patient's response to the doctor's query:
        {patient_response}
```

Supplementary Method 14. Questionnaire Design for Medical Student User Study
To systematically evaluate the performance of AIPatient in comparison to H-SPs, we developed a structured questionnaire encompassing key domains of fidelity, usability, and educational effectiveness. The design was informed by established frameworks in medical simulation and human-computer interaction (HCI). Fidelity metrics such as role adherence, emotional realism, and contextual appropriateness were adapted from prior work in simulated patient methodology, which emphasizes the importance of consistency, believability, and alignment with clinical scenarios in virtual and human actors[8,9]. Usability items, including ease of interaction and technical reliability, drew from validated HCI instruments such as the System Usability Scale[10] and conversational agent design literature focusing on naturalistic dialogue and response relevance[9]. Effectiveness was assessed through perceived diagnostic accuracy and learner satisfaction, drawing on best practices in simulation-based assessment and educational outcomes research[11]. In addition, an OSCE-style checklist was embedded to evaluate whether key components of history-taking (e.g., chief complaint, medication review, psychosocial context) were elicited, following classical OSCE protocols[12]. This multi-dimensional instrument enabled a comprehensive comparison of AIPatient and H-SPs grounded in both educational theory and user experience research.The final questionnaire used in medical student user study is available in **Supplementary Table 13**.

Supplementary Discussion

Supplementary Discussion 1. Benchmarking computational efficiency and operational cost of the AIPatient System

To evaluate the computational efficiency of the AIPatient system, we conducted a benchmarking analysis of cost and response time across several large language models, using the average performance over five representative clinical questions selected from the gold standard QA dataset (**Supplementary Discussion Table 1**). Proprietary models such as Claude-3-Haiku, GPT-3.5-Turbo, and GPT-4o demonstrated the lowest per-question costs (ranging from <$0.01 to $0.02) and fastest response times (6.28–7.55 seconds), making them attractive options for real-time applications. In contrast, open-source models such as Deepseek-v3-671b and Llama-4-16b exhibited substantially higher latency, with average response times exceeding one minute (e.g., 140.74 seconds for Deepseek-v3-671b), limiting their practical use in interactive settings despite their accessibility. When jointly considering speed, cost, and accuracy, GPT-4-Turbo emerged as the most balanced and effective model. It achieved a relatively low response time (9.37 seconds) and moderate cost ($0.07 per question), while outperforming all other models in QA accuracy (94.15%). These findings support the selection of GPT-4-Turbo as the optimal model for powering the AIPatient system, enabling accurate, efficient, and scalable deployment for clinical simulation and training.

Supplementary Discussion Table 1 Cost Analysis Per Question by Model

Model Name	Total Cost (USD)	Time Elapsed (s)	Total Input Tokens	Total Output Tokens
Claude-3-Haiku	0.01	7.55	6720.40	651.20
Claude-3.5-Sonnet	0.02	17.14	7602.60	535.60
Claude-4-Sonnet	0.03	21.26	8586.20	694.60
Claude-4-Opus	0.12	18.85	8199.00	569.00
GPT-3.5-Turbo	<0.01	6.94	7277.60	488.60
GPT-4o	0.02	6.28	7084.60	493.20
GPT-4-Turbo	0.07	9.37	6416.00	391.60
Deepseek-v3-671b	0.01	140.74	7269.70	7799.10
Llama-4-16b	-	64.53	7657.30	803.10
Qwen-3-32b	-			

Note: This table summarizes the average cost, response time, and token usage per question across different large language models. The values were computed by aggregating results from 5 clinical questions. Costs are reported in USD per question. Token counts reflect both input and output usage. Missing values indicate unavailable or unsupported cost data for open-source models.

Supplementary Discussion 2. Additional User Study Results

Quantitative Evaluation: Questionnaire Analysis

The quantitative findings from the user study offer a detailed comparison between AIPatient and H-SPs in terms of both information elicitation and user-perceived interaction quality. To ensure a fair comparison, the user interface was kept identical for both AIPatient and human-simulated patient conditions. In both cases, students interacted via the same chat-based interface, presented with turn-based dialogue and consistent formatting (**Supplementary Fig. 7-8**).

Fidelity

In terms of role and text adherence, the AIPatient slightly outperformed H-SPs on both items: students agreed that the AIPatient more consistently followed the case script (mean = 4.34 vs. 4.21) and better matched the intended medical condition (4.24 vs. 3.90, $p < 0.1$). With respect to contextual appropriateness, AIPatient were rated higher on the naturalness of responses (4.20 vs. 3.95), more coherent (4.32 vs. 4.08 and clinically relevant (4.27 vs. 4.23) in their answers. The most pronounced difference in favor of the AIPatient was observed in emotional realism, where students rated the AIPatient significantly higher than the H-SPs (4.37 vs. 3.47, $p < 0.01$). This finding suggests that, through affective modeling, the AIPatient was more effective at conveying simulated emotions such as anxiety or distress—an important element in psychiatric and primary care training scenarios.

Usability

Participants reported that interacting with the AIPatient requires less effort (4.20 vs. 3.79) and was more technically reliable (4.39 vs. 3.79, $p < 0.01$). This points to the AI system's ability to deliver a smooth and responsive experience that is less prone to human delay or deviation. Students also agreed that the AIPatient could be readily integrated into training programs (4.02 vs. 3.92), further supporting its feasibility in real-world medical education settings.

Effectiveness

AIPatient were rated more favorably on both outcome measures in the training effectiveness metric. Students indicated that the AIPatient sessions more often allowed them to reach a preliminary diagnosis (4.27 vs. 3.87) and were more helpful in improving clinical reasoning skills (4.41 vs. 3.97, $p < 0.05$). These modest yet consistent advantages suggest that AIPatient may be particularly well suited for structured, formative training environments where diagnostic accuracy and cognitive integration are central learning objectives.

Analysis of the OSCE-style checklist, which tracked whether students successfully collected key clinical information, showed that AIPatient performed comparably—and in several areas better—than human-simulated patients. AIPatient achieved higher completion rates in chief complaints (97.5% vs. 94.8%), relevant medical history (100% vs. 89.7%), and medication/allergy review (100% vs. 87.1%). The largest advantage was seen in psychosocial context (87.8% vs. 64.1%), suggesting stronger contextual responsiveness. Overall, AIPatient demonstrated strong and consistent performance across clinical documentation tasks.

In summary, the quantitative data demonstrate that AIPatient matched or exceeded H-SPs across most evaluated dimensions. While certain aspects—such as natural conversational tone—remain strengths of human actors, the AIPatient's consistency, emotional expressiveness, and reliability position it as a compelling alternative for scalable, standardized medical education.

Qualitative Evaluation: Structured Interview Findings

A thematic analysis of post-interaction interviews with participants revealed several themes related to the fidelity, usability, and effectiveness of the AIPatient system as a tool for clinical education.

Fidelity

Participants generally found the interactions with AIPatient to be realistic and pedagogically valuable. The AI's consistent emotional tone and role-specific personality traits (e.g., repetitive speech, concern over lifestyle factors) were viewed as aligning with plausible patient presentations. In some cases, AIPatient was described as more emotionally engaged than its human counterpart, effectively simulating complex patient behaviors. However, concerns were raised regarding overly suggestive or leading responses, which may inadvertently bias student inquiry. Several students also noted the need for broader diagnostic diversity and improved responsiveness to non-standard questioning.

Usability

Participants consistently emphasized the efficiency of the AI-driven simulated patient, noting that the system responded significantly faster than H-SPs. This rapid response time allowed for more streamlined interactions and potentially greater exposure to diverse clinical cases within a constrained time frame. However, several participants reported that the AIPatient's responses were occasionally overly verbose or repetitive. While this verbosity was partly attributable to character design (e.g., simulating a talkative or anxious patient), it occasionally led to cognitive overload and reduced conversational clarity. Suggestions for improvement included implementing more concise response strategies and improving alignment between student prompts and AIPatient outputs.

Effectiveness

Students generally perceived the AIPatient as an effective tool for supporting clinical skills training, particularly in the domain of medical history taking. The AIPatient's emotional expressiveness—particularly in simulating symptoms such as anxiety or depression—was highlighted as a strength, providing a realistic portrayal of patient affect that challenged students to adapt their questioning and empathic responses. Several participants noted that the AIPatient helped reinforce communication strategies and clinical reasoning, particularly for junior students in the early stages of clinical education. Nevertheless, some interviews revealed limitations in the AIPatient's ability to provide nuanced or complete clinical information. Missing details regarding symptom chronology, precipitating factors, or modifiers were noted, and participants expressed a desire for richer and more structured case narratives to support comprehensive history-taking.

In summary, students perceived the AIPatient as a usable, engaging, and high-fidelity simulation platform with strong potential for medical education. While several areas for refinement were identified—including tailoring response length, enhancing case complexity, and improving flexibility in dialogue—the system was broadly regarded as a promising adjunct to traditional training modalities.

Supplementary Tables

Supplementary Table 1 Patient demographics and clinical characteristics

Medical Entity	Attributes	Categories	Overall
Patient	Number of Patients		1500
	GENDER, n (%)	F	608 (40.5)
		M	892 (59.5)
	AGE, mean (SD)		51.0 (18.3)
	ETHNICITY, n (%)	Asian	55 (3.7)
		Black/African American	128 (8.5)
		Hispanic/Latino	71 (4.7)
		Other	252 (16.8)
		White	994 (66.3)
	RELIGION, n (%)	Christian	592 (39.5)
		Non-Christian Religions	88 (5.9)
		Other/Unspecified	820 (54.7)
	MARITAL_STATUS, n (%)	Married	742 (49.5)
		Other/Unspecified	128 (8.5)
		Single	630 (42.0)
Admissions	DURATION, mean (SD)		4.5 (4.6)
	ADMISSION_TYPE, n (%)	Emergency	1,435 (95.7)
		Urgent	65 (4.3)
	ADMISSION_LOCATION, n (%)	Emergency Admission	916 (61.1)
		Referral	296 (19.7)
		Transfer	288 (19.2)
	DISCHARGE_LOCATION, n (%)	Facility	172 (11.5)
		Home	1,183 (78.9)
		Hospice	5 (0.3)
		Other	112 (7.5)
		Transfer	28 (1.9)
	INSURANCE, n (%)	Government	100 (6.7)
		Medicaid	116 (7.7)
		Medicare	331 (22.1)
		Private	894 (59.6)
		Self Pay	59 (3.9)
Symptoms	Number of Symptoms, n (%)		11,013
		Denied Symptom	3,062 (27.8)
		Symptom with Duration	176 (1.6)
		Symptom with Frequency	472 (4.3)
		Symptom with Intensity	630 (5.7)
Allergies	Number of Allergies		598
Medical History	Number of Medical History		3,899
Family History	Number of Family Members		976
	Number of Family Medical History		981
Social History	Number of Social History		4,537

Supplementary Table 2 AIPatient KG Nodes and Edges Statistics

Nodes	Count	Relationships	Counts
Patient	1495		
Admission	1500	HAS_ADMISSION	1500
Symptom	3955	HAS_SYMPTOM	7000
		HAS_NOSYMPTOM	2971
Duration	683	HAS_DURATION	1544
Intensity	206	HAS_FREQUENCY	383
Frequency	190	HAS_INTENSITY	408
History	2485	HAS_MEDICAL_HISTORY	3865
Vital	717	HAS_VITAL	2425
Allergy	227	HAS_ALLERGY	596
Social History	3287	HAS_SOCIAL_HISTORY	4497
Family Member	143	HAS_FAMILY_MEMBER	885
Family Medical History	553	HAS_MEDICAL_HISTORY	808

Supplementary Table 3 NER F1 by Entity Categories

Entity Categories	claude-3 haiku	claude-3 sonnet	claude-3.5 sonnet	claude-4-sonnet	claude-4-opus	gpt-3.5 turbo	gpt-4o	gpt-4 turbo	Deepseek -v3-671b	Qwen -3-32b	Llama -3-70b
Symptom Group[1]	0.68	0.69	0.7	0.69	0.7	0.69	0.74	**0.87**	0.67	0.69	0.66
Medical History	0.69	0.7	0.78	0.77	0.77	0.72	0.75	**0.9**	0.75	0.73	0.76
Allergies	0.69	0.69	0.89	0.9	0.89	0.87	0.96	**0.98**	0.96	0.67	0.67
Family and Social History Group[2]	0.71	0.71	0.75	0.75	0.76	0.71	0.74	**0.91**	0.73	0.73	0.77
Total	0.69	0.7	0.73	0.73	0.73	0.7	0.75	**0.89**	0.71	0.7	0.7

[1]Symptom Group includes symptoms (both symptoms and denied symptoms), duration, intensity and frequency.
[2]Family and Social History Group includes Family History (family members and their medical history) and Social History.

Supplementary Table 4 NER Precision by Entity Categories

Entity Categories	claude-3 haiku	claude-3 sonnet	claude-3.5 sonnet	claude-4-sonnet	claude-4-opus	gpt-3.5 turbo	gpt-4o	gpt-4 turbo	Deepseek-v3-671b	Qwen-3-32b	Llama-3-70b
Symptom Group[1]	0.78	0.82	0.81	0.65	0.66	0.92	0.76	0.92	0.61	0.71	**0.93**
Medical History	0.89	0.81	0.85	0.86	0.85	0.86	0.81	**0.98**	0.8	0.79	0.81
Allergies	**1**	**1**	0.86	0.95	0.95	0.92	0.95	0.96	0.95	**1**	**1**
Family and Social History Group[2]	0.89	0.86	0.78	0.75	0.76	0.9	0.79	**0.96**	0.75	0.74	0.82
Total	0.83	0.83	0.81	0.71	0.73	0.9	0.78	**0.94**	0.68	0.74	0.87

[1]Symptom Group includes symptoms (both symptoms and denied symptoms), duration, intensity and frequency.
[2]Family and Social History Group includes Family History (family members and their medical history) and Social History

Supplementary Table 5 NER Recall by Entity Categories

Entity Categories	claude-3 haiku	claude-3 sonnet	claude-3.5 sonnet	claude-4-sonnet	claude-4-opus	gpt-3.5 turbo	gpt-4o	gpt-4 turbo	Deepseek -v3-671b	Qwen -3-32b	Llama -3-70b
Symptom Group[1]	0.61	0.59	0.62	0.74	0.74	0.55	0.72	**0.82**	0.75	0.66	0.51
Medical History	0.56	0.62	0.72	0.7	0.71	0.62	0.69	**0.83**	0.71	0.68	0.72
Allergies	0.53	0.53	0.92	0.86	0.83	0.82	0.98	**1**	0.98	0.5	0.5
Family and Social History Group[2]	0.59	0.61	0.72	0.75	0.76	0.59	0.69	**0.86**	0.71	0.71	0.72
Total	0.59	0.6	0.67	0.74	0.74	0.58	0.71	**0.84**	0.74	0.67	0.59

[1]Symptom Group includes symptoms (both symptoms and denied symptoms), duration, intensity and frequency.
[2]Family and Social History Group includes Family History (family members and their medical history) and Social History

Supplementary Table 6 System performance on the non-differentiating set of medical record categories (GPT-4-Turbo)

Few Shot	Retrieval Agent	Abstraction Agent	Admission	Patient	Allergy	Vitals
✓	✓	✓	**100.00%**[1]	**100.00%**	**96.67%**	**94.74%**
✓	✓		**100.00%**	**100.00%**	**96.67%**	**94.74%**
✓		✓	**100.00%**	**100.00%**	**96.67%**	**94.74%**
✓			**100.00%**	**100.00%**	**96.67%**	**94.74%**
	✓	✓	**100.00%**	**100.00%**	**96.67%**	**94.74%**
	✓		**100.00%**	**100.00%**	**96.67%**	**94.74%**
		✓	**100.00%**	**100.00%**	90.00%	**94.74%**
Only with *KG Query Generation Agent*			**100.00%**	98.28%	**96.67%**	**94.74%**
Without *Reasoning RAG* & Without *AIPatient KG*			100.00%	87.50%	48.08%	90.00%

[1] Highest accuracy in each category is in bold.

**Supplementary Table 7** One-way ANOVA (two-sided) Results for Robustness Evaluation on Three Paraphrase Sets				
**Entity Category**	**Sum of Squares**	**Degree of Freedom**	**F**	**P-Value**
Overall				
Paraphrase Group	0.0818	3	0.6126	0.5420
Residual	102.9382	1541		
Symptom				
Paraphrase Group	0.0821	3	0.54	0.5835
Residual	16.4383	216		
Medical History				
Paraphrase Group	1.7160	3	5.3038	0.00589
Residual	25.7222	1596		
Family and Social History				
Paraphrase Group	0.0896	3	0.2536	0.7761
Residual	41.7094	235		

Supplementary Table 8 Two-Sample T-test of Accuracy Difference Between Paraphrase Question and Original Question[1]

Question Category	Original Proportion	Paraphrase Proportion	Difference	Z=Statistics	P-Value
Paraphrase 1					
Overall	94.15%	93.79%	0.19%	0.13	0.90
Symptom	91.20%	93.15%	1.37%	-0.29	0.77
Medical History	87.10%	92.59%	3.70%	-0.61	0.54
Family and Social History	85.56%	75.00%	10.00%	1.58	0.11
Paraphrase 2					
Overall	94.15%	92.04%	1.94%	1.22	0.22
Symptom	91.20%	93.15%	1.37%	-0.29	0.77
Medical History	87.10%	68.52%	18.52%	2.31	0.02
Family and Social History	85.56%	77.50%	7.50%	1.22	0.22
Paraphrase 3					
Overall	94.15%	92.61%	1.55%	0.99	0.32
Symptom	91.20%	89.04%	2.74%	0.53	0.60
Medical History	87.10%	74.07%	14.81%	1.91	0.06
Family and Social History	85.56%	79.75%	6.25%	1.03	0.30

[1] Z score and P-values reports two sample t-test (two tailed) on the proportional difference between original cypher query evaluation questions and paraphrased questions. Significance at 0.01 level and no statistical significant differences are identified.

Supplementary Table 9 One-way ANOVA Results (two-sided) for Stability Evaluation on 32 Personality Groups

Entity Category	Sum of Squares	Degree of Freedom	F	P-Value
Overall				
Paraphrase Group	0.3529	31	0.7820	0.7990
Residual	23.2941	1600		
Symptom				
Paraphrase Group	1.4023	31	1.1104	0.3230
Residual	9.1250	224		
Family and Social History[1]				
Paraphrase Group	0.9023	31	0.6774	0.9024
Residual	9.6250	224		

[1] For the Medical History category, all personality groups have 0% data loss proportion; no statistical test is conducted due to lack of variation.

Supplementary Table 10 System performance on the medical record categories in CORAL

Few Shot	Retrieval Agent	Abstraction Agent	Overall	Symptom Group	Medical History	Family and Social History
✓	✓	✓	**81.04%**[1]	**100.00%**	**71.55%**	62.40%
✓	✓		72.04%	93.33%	66.38%	61.60%
✓		✓	66.82%	**100.00%**	41.38%	**64.00%**
✓			65.40%	**100.00%**	32.76%	61.60%
	✓	✓	62.09%	66.67%	56.03%	60.00%
	✓		69.67%	60.00%	57.76%	61.60%
		✓	63.27%	80.00%	37.07%	61.60%
Only with *KG Query Generation Agent*			60.43%	80.00%	28.45%	63.20%

[1] Highest accuracy in each category is in bold.

Supplementary Table 11 One-way ANOVA (two-sided) Results for Robustness Evaluation on Three Paraphrase Sets in CORAL

Entity Category	Sum of Squares	Degree of Freedom	F	P-Value
Overall				
Paraphrase Group	0.2954	3	0.5924	0.6200
Residual	256.3830	1542		

Supplementary Table 12 One-way ANOVA (two-sided) Results for Stability Evaluation on 32 Personality Groups in CORAL

Entity Category	Sum of Squares	Degree of Freedom	F	P-Value
Overall				
Paraphrase Group	0.7044	31	0.65140	0.9308
Residual	93.7647	2688		

Supplementary Table 13 Questionnaire for Comparing AIPatient vs. H-SPs

Metric	Question	1	2	3	4	5
Fidelity						
Role/Text Adherence	The SP followed the case script without contradictions.					
	The SP's responses matched the intended medical condition.					
Contextual Appropriateness	The SP's responses felt natural and relevant to my questions.					
Emotional Realism	The SP displayed believable emotions (e.g., pain, anxiety).					
Coherence/Consistency	The SP's dialogue was coherent (no abrupt shifts).					
Response Quality	The SP's answers were directly relevant to clinical questions.					
Usability						
Ease of Use	Interacting with this SP required minimal effort.					
	I encountered no technical difficulties (e.g., delays).					
Feasibility/Scalability	This SP could be easily integrated into our training program.					
Effectiveness						
Diagnostic Accuracy	I have reached a preliminary diagnosis at the end.					
OSCE Checklist						
	Chief complaint (duration/severity)					
	Relevant medical history					
	Medication/allergy review					
	Psychosocial context					
	Review of systems (ROS)					
Learner Satisfaction	This session improved my clinical reasoning skills.					

Instructions:

- For Likert-scale items, score: **1 = Strongly Disagree, 2 = Disagree, 3 = Neutral, 4 = Agree, 5 = Strongly Agree.**
- For OSCE checklist, write a check if this information is collected.

Supplementary Figures

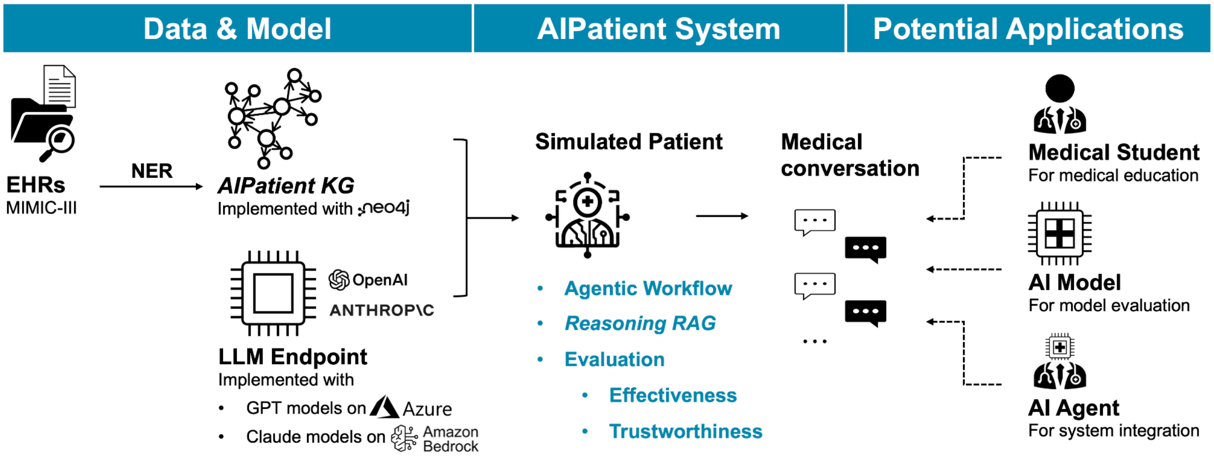

Supplementary Fig. 1. AIPatient as an integrative system with EHR-based knowledge input, LLM endpoints as preprocessing and reasoning models, and potential applications. Created by the authors using built-in Microsoft PowerPoint icons and shapes.

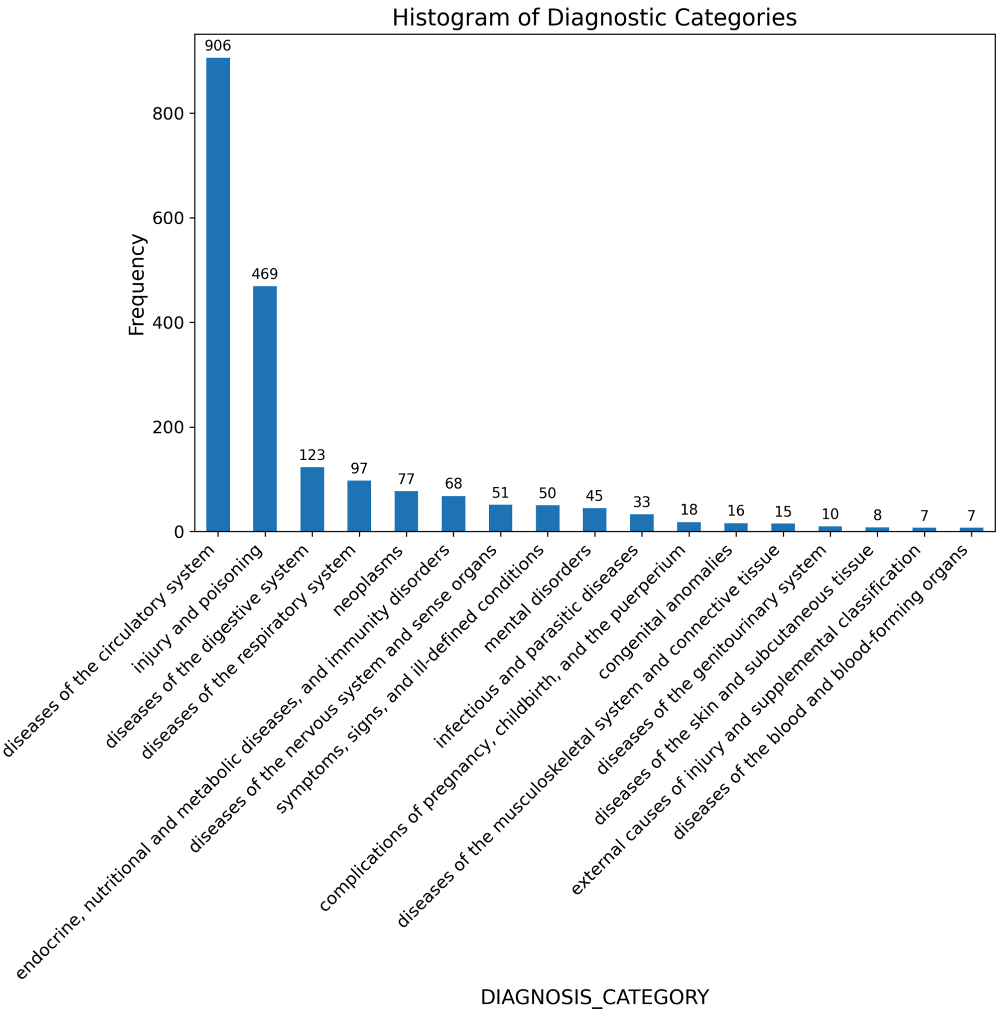

Supplementary Fig. 2. AIPatient KG's distribution of patients in each major diagnostic category. We also restrict the current analysis scope to patients aged 18 years and above and exclude cases of parturition, elective procedures, severe car accidents, or instances where the patient lost the ability to communicate or interact with medical care personnel. These cases are excluded because they involve clinical complexities that fall outside the intended focus of AIPatient's patient-provider interaction model. Patients unable to communicate effectively are incapable of providing disease-related information to medical professionals. Similarly, including patients under 18 would introduce unique clinical and developmental complexities requiring a separate, pediatric-focused model. Cases like parturition, elective procedures, and severe car accidents also involve highly specialized care and specific clinical pathways that add complexity beyond the system's current scope.

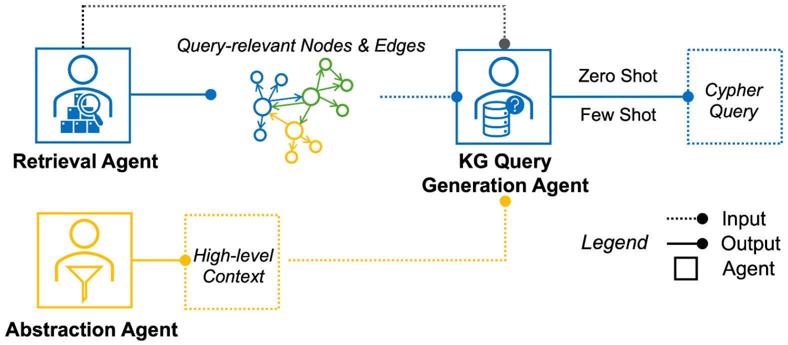

Supplementary Fig. 3. Ablation studies data flow for Cypher query evaluation. Created by the authors using built-in Microsoft PowerPoint icons and shapes.

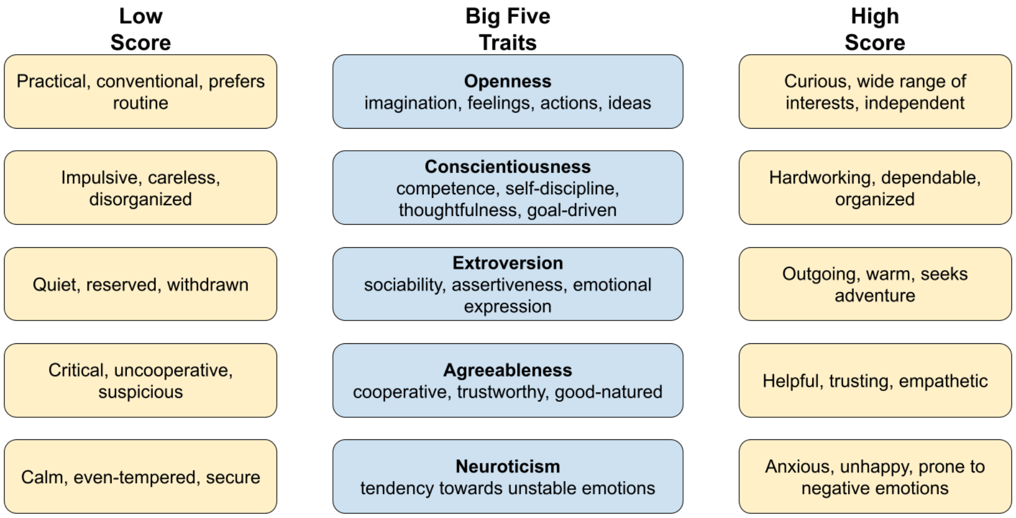

Supplementary Fig. 4. Big Five Personality describes human personality using five broad domains, each with a spectrum from low to high scores. To simulate personality profiles within the AIPatient system, we generated 32 unique profiles by randomly assigning either a high or low score for each of the five personality dimensions. Each trait was independently assigned, leading to 32 unique combinations.

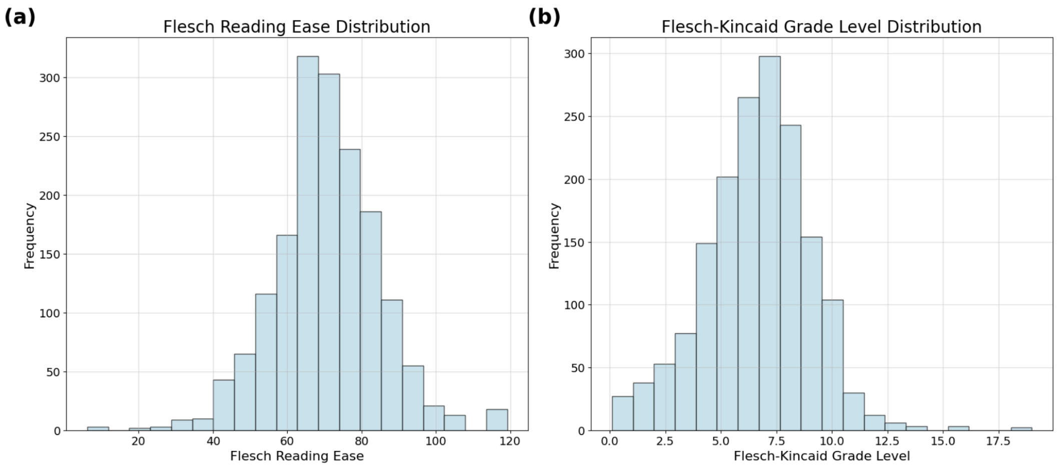

Supplementary Fig. 5. Distribution of the Flesch Reading Ease and the Flesch-Kincaid Grade Level in AIPatient outputs in CORAL

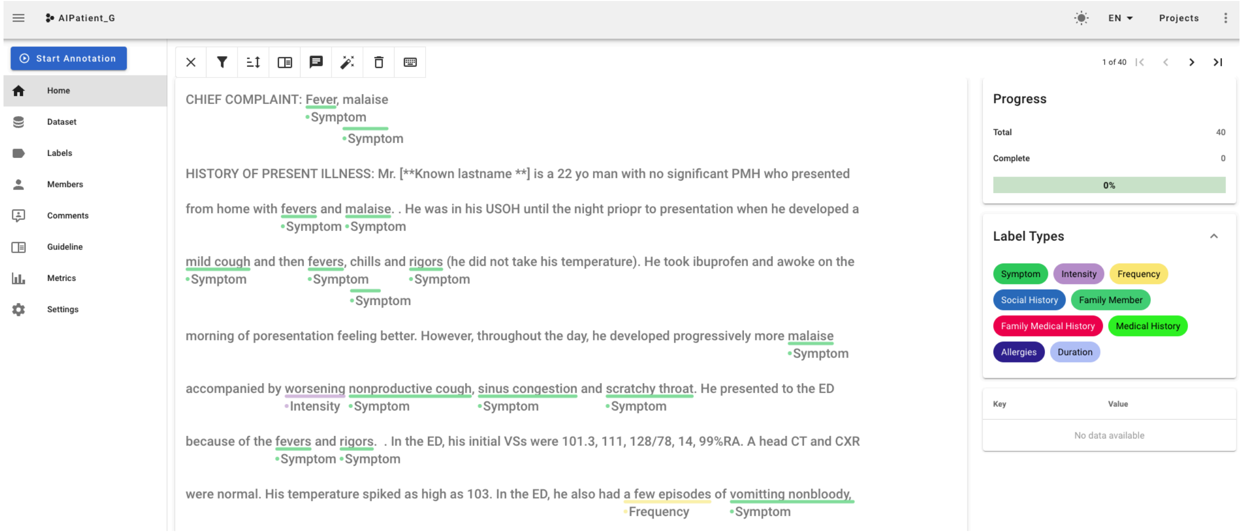

Supplementary Fig. 6. Annotation Interface with Doccano. The MDs used the Doccano platform for NER annotation [13]. MDs were provided with predefined categories (e.g., symptoms, medical history, allergies) and marked relevant portions of the text that corresponded to these categories.

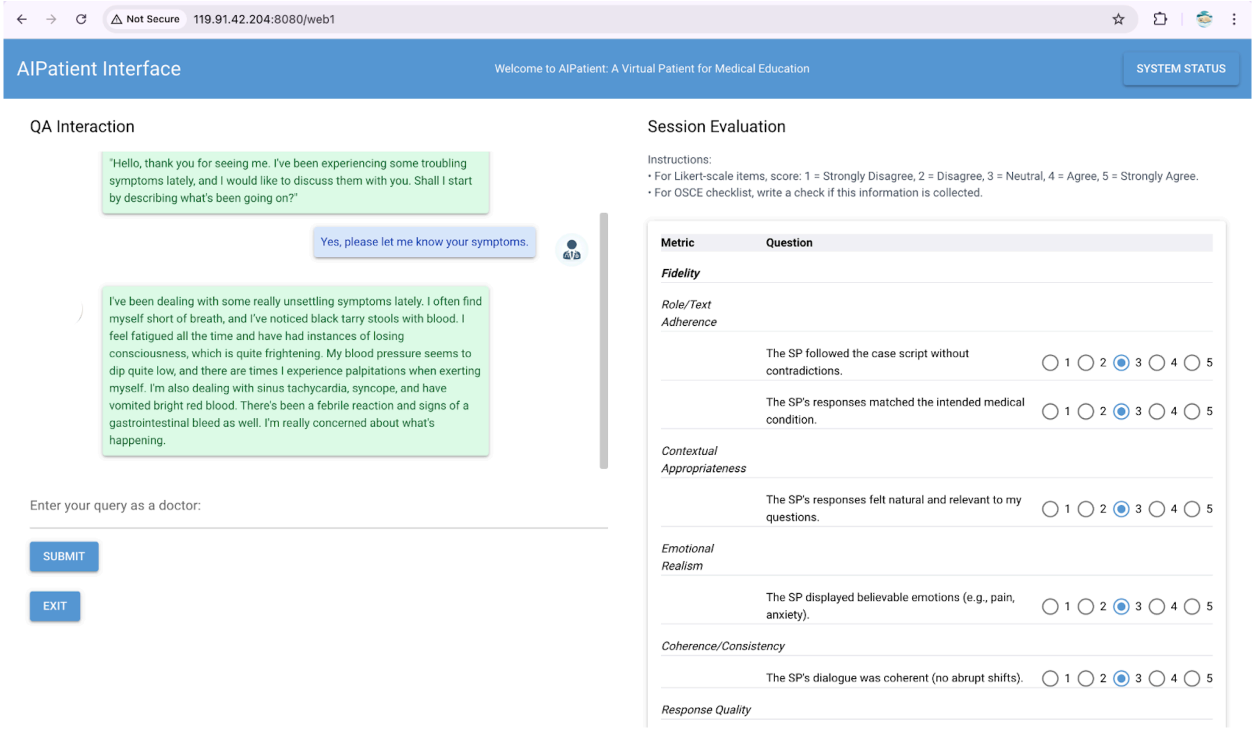

Supplementary Fig. 7. User study interface, medical student panel. This interface displays a real-time conversation between a medical student and the AIPatient or H-SPs, along with an integrated session evaluation panel. Users assess simulated patient performance across fidelity, emotional realism, and response coherence using Likert-scale and OSCE-style checklist metrics.

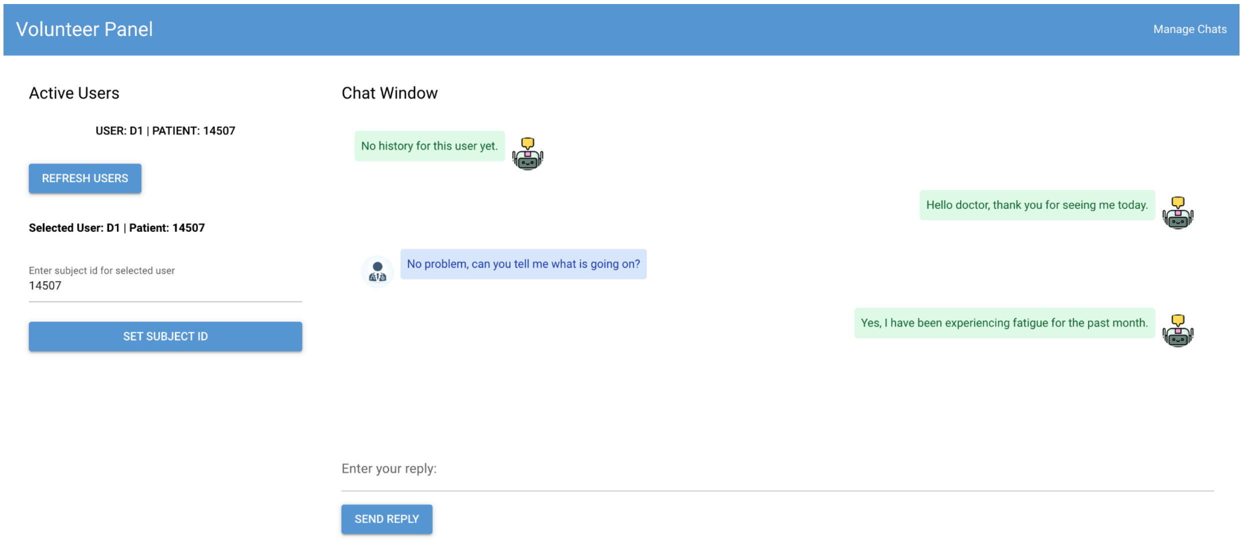

Supplementary Fig. 8. User study interface, volunteer H-SPs panel. This interface allows volunteers to act as simulated patients during clinical training sessions. The panel displays active users, enables case assignment via subject ID, and supports real-time chat interactions between the volunteer and trainee through a unified text-based window.

References

1. Kong, A. *et al.* Better zero-shot reasoning with role-play prompting. in *Proceedings of the 2024 Conference of the North American Chapter of the Association for Computational Linguistics: Human Language Technologies (Volume 1: Long Papers)* (Association for Computational Linguistics, Stroudsburg, PA, USA, 2024). doi:10.18653/v1/2024.naacl-long.228.
2. Anthropic. Giving Claude a role with a system prompt. *Anthropic* https://docs.anthropic.com/en/docs/build-with-claude/prompt-engineering/system-prompts.
3. Brown, T. B. *et al.* Language models are few-shot learners. in *Proceedings of the 34th International Conference on Neural Information Processing Systems* (Curran Associates Inc., Red Hook, NY, USA, 2020).
4. Anthropic. Use XML tags to structure your prompts. *Anthropic* https://docs.anthropic.com/en/docs/build-with-claude/prompt-engineering/use-xml-tags.
5. Neo4j, Inc. NEO4J GRAPH DATA PLATFORM | Blazing-Fast Graph, Petabyte Scale. *Neo4j* https://neo4j.com/.
6. Zheng, H. S. *et al.* Take a Step Back: Evoking Reasoning via Abstraction in Large Language Models. in *The Twelfth International Conference on Learning Representations* (2024).
7. Goldberg, L. R. An alternative 'description of personality': the big-five factor structure. *J. Pers. Soc. Psychol.* **59**, 1216–1229 (1990).
8. Nestel, D. & Bearman, M. *Simulated Patient Methodology: Theory, Evidence and Practice*. (Wiley-Blackwell, Chichester, England, 2014).
9. Jarvis, M. P., Nuzzo-Jones, G. & Heffernan, N. T. Applying machine learning techniques to rule generation in intelligent tutoring systems. in *Intelligent Tutoring Systems* 541–553 (Springer Berlin Heidelberg, Berlin, Heidelberg, 2004).

10. Brooke, J. SUS-A Quick and Dirty Usability Scale. in *Usability Evaluation in Industry* (eds. Jordan, P. W., Thomas, B., Weerdmeester, B. A. & Mcclelland, I. L.) 189–194 (Taylor & Francis, London, 1996).
11. Cook, D. A. & Hatala, R. Validation of educational assessments: a primer for simulation and beyond. *Adv. Simul.* **1**, 31 (2016).
12. Harden, R. M. & Gleeson, F. A. Assessment of clinical competence using an objective structured clinical examination (OSCE). *Med. Educ.* **13**, 41–54 (1979).
13. doccano. doccano. *github* https://github.com/doccano/doccano.
